# Adoption and maintenance of gym-based strength training in the community setting in adults with excess weight or type 2 diabetes: a randomized controlled trial

**DOI:** 10.1186/s12966-015-0266-5

**Published:** 2015-08-25

**Authors:** Megan Teychenne, Kylie Ball, Jo Salmon, Robin M. Daly, David A Crawford, Parneet Sethi, Michelle Jorna, David W. Dunstan

**Affiliations:** Deakin University, Centre for Physical Activity and Nutrition Research, School of Exercise and Nutrition Sciences, Melbourne, Victoria, Australia; Baker IDI Heart and Diabetes Institute, Melbourne, Australia; School of Sport Science, Exercise and Health, The University of Western Australia, Perth, Australia; Cancer Prevention Research Centre, School of Population Health, The University of Queensland, Brisbane, Australia; Department of Epidemiology and Preventive Medicine, Monash University, Melbourne, Australia

**Keywords:** Strength training, Behavioral counselling, Intervention, Adherence, Type 2 diabetes

## Abstract

**Background:**

Participant adoption and maintenance is a major challenge in strength training (ST) programs in the community-setting. In adults who were overweight or with type 2 diabetes (T2DM), the aim of this study was to compare the effectiveness of a standard ST program (SST) to an enhanced program (EST) on the adoption and maintenance of ST and cardio-metabolic risk factors and muscle strength.

**Methods:**

A 12-month cluster-randomized controlled trial consisting of a 6-month adoption phase followed by a 6-month maintenance phase. In 2008–2009, men and women aged 40–75 years (*n* = 318) with T2DM (*n* = 117) or a BMI >25 (*n* = 201) who had not participated in ST previously were randomized into either a SST or an EST program (which included additional motivationally-tailored behavioral counselling). Adoption and maintenance were defined as undertaking ≥ 3 weekly gym-based exercise sessions during the first 6-months and from 6–12 months respectively and were assessed using a modified version of the CHAMPS (Community Healthy Activity Models Program for Seniors) instrument.

**Results:**

Relative to the SST group, the adjusted odds ratio (OR) of adopting ST for all participants in the EST group was 3.3 (95 % CI 1.2 to 9.4). In stratified analyses including only those with T2DM, relative to the SST group, the adjusted OR of adopting ST in the EST group was 8.2 (95 % CI 1.5–45.5). No significant between-group differences were observed for maintenance of ST in either pooled or stratified analyses. In those with T2DM, there was a significant reduction in HbA1c in the EST compared to SST group during the adoption phase (net difference, -0.13 % [-0.26 to -0.01]), which persisted after 12-months (-0.17 % [-0.3 to -0.05]).

**Conclusions:**

A behaviorally-focused community-based EST intervention was more effective than a SST program for the adoption of ST in adults with excess weight or T2DM and led to greater improvements in glycemic control in those with T2DM.

**Trial registration:**

Registered at ACTRN12611000695909 (Date registered 7/7/2011).

## Background

Strength training (ST) or progressive resistance training has been shown to be a safe and effective treatment strategy for improving glycemic control in people with or at risk of type 2 diabetes (T2DM) [1–4], including those who are overweight [1]. In recognition of the benefits of strength training, a community-based ST program, termed ‘*Lift for Life*’® (http://www.liftforlife.com.au), has been implemented in Australia for adults with T2DM and those with certain risk factors (e.g. overweight/obese). The ‘*Lift for Life*’® program has been employed within existing community health and fitness facilities through the development of a network of accredited providers in Australian cities.

It is estimated that 64 % of overweight adults [5] and 60 % of adults with T2DM in Australia [6] and the US [7] are not sufficiently active, which is considerably more than the general population [8] despite reports showing few differences in reported barriers to physical activity between those with and without abnormal glucose metabolism [9]. Long-term maintenance of physical activity is paramount to sustain improvements in glycemic control; however, promoting the adoption and maintenance of ST programs has been identified as a major challenge [2]. Thus, there is a need for research to identify effective approaches to optimise adoption and maintenance to community-based ST programs for people with or at risk of T2DM [10].

Studies in older adults have shown that matching an intervention to a participant’s level of motivational readiness [11] is more effective for increasing and maintaining physical activity levels than a one-treatment-fits-all approach [12]. Further, studies in overweight adults [13] and adults with T2DM [14, 15] have shown that interventions involving physical activity counselling, based on principles from the social cognitive theory (SCT) [16] (e.g. social support and self-efficacy) are more effective for promoting and maintaining physical activity levels than usual care treatment. Thus the current study drew on the *Lift for Life* ® ST model and the theoretical framework of the SCT [17], as well as principles of the stage of readiness for behavior change [11]. In adults who were overweight or with T2DM, the aim of this study was to compare the effectiveness of a Standard group-based ST (SST) program to an Enhanced ST (EST) program (i.e. standard ST plus the addition of behavioral counselling) in regards to: 1) adoption and maintenance of ST; and 2) long-term changes in cardio-metabolic risk factors (adiposity), muscle strength and glycemic control. For the purpose of this study, adoption was defined as participating in the *Lift for Life* ® regular ST program at least 3 times per week during the first 6-months, whilst maintenance was defined as ongoing participation (at least 3 sessions per week) in the program from 6–12 months.

## Methods

### Study design

This was a 12-month cluster-randomized controlled trial, termed Strength TRaining ONGoing (STRONG study), comprising a 6-month adoption phase followed by a 6-month maintenance phase. Repeated assessments were performed at baseline, 6- and 12-months. A convenience sample of health and fitness centres from across metropolitan Melbourne and Geelong, Victoria Australia (*n* = 14) were approached and agreed to provide access to their leisure facility for the ST program (based on the commercial *Lift for Life* program®) to be undertaken by participants enrolled in the study at a reduced cost (range $36–$60 per month). Qualified exercise training staff were recruited for the STRONG program and were responsible for overseeing the exercise intervention. A cluster randomised approach was used to minimise the potential for contamination across individuals within centres. The study was approved by the Deakin University Ethics Committee and by the Baker IDI Heart and Diabetes Institute Ethics Committee.

### Participants

In accordance with the target audience of the *Lift for Life* program®, men and women aged 40–75 years old who had T2DM (>3-months) and not currently performing ST were recruited. Due to initial recruitment difficulties, the target group was expanded to also include those who were classified as being overweight [BMI >25]) and not currently performing ST, on the grounds that being overweight is recognised as a major risk factor for developing T2DM [18]. Multiple recruitment methods were used to identify participants, including a local letterbox drop incorporating a flyer advertising the research project; a launch at each facility; advertisements placed in diabetes educators’ and GP’s waiting rooms; and advertisements placed in state and local newspapers. Interested participants were screened by telephone or during information sessions held at the facilities and were included in the study once they confirmed that they had medical clearance from their physician and were willing to participate as a fee-paying member.

**Fig. 1:**
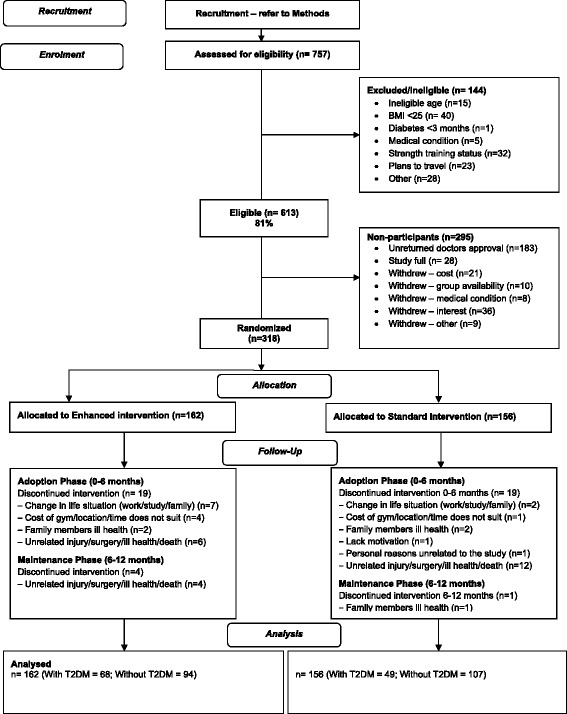
STRONG participant flowchart

Participants were excluded based on the following criteria: those with uncontrolled hypertension and/or physical conditions that precluded participation in ST; a medical condition listed in the ACSM absolute exercise contraindications [19]; or plans to travel for four or more consecutive weeks over the 12-month study period. For those screened over the phone, if self-reported BMI was between 24 and 26, a home visit was arranged to confirm BMI status to determine eligibility. Initial sample size calculations indicated that 256 participants (128 in each intervention arm) were required to detect a difference between groups of 50-min of ST per week during the adoption and maintenance phases (alpha = 0.05, power = 0.80, ICC = 0.1) assuming an estimated 15 % loss to follow-up. This was based on a continuous outcome. However, analyses used a dichotomous outcome (sessions per week) since the specific recommendations of the *Lift for Life*® program were to undertake two supervised and one unsupervised ST session per week. Of the interested participants (*n* = 757), 613 met the full entry criteria and 318 (187 men and 131 women) agreed to participate (Fig. 1). All participants provided informed written consent.

### Randomization

To minimise contamination, randomization was performed by the project manager at the Health and Fitness facility level in the order of receipt of organisational consent. Block randomization was conducted by an independent researcher using Microsoft Excel. Participants were able to choose which fitness centre they would attend, however, they did not have prior knowledge of which intervention arm the centre had been allocated to until after they had commenced their program and baseline measures were completed. Exercise training staff were blinded to the group assessment.

### Standard intervention

Participants in the SST intervention group (*n* = 156) followed an initial eight-week program, based on the *Lift for Life* program® [20]. Participants followed the *Lift for Life* program® principles across all of the health centres involved. Twice weekly (45–60 mins) supervised group exercise sessions were provided and participants were encouraged to engage in one additional unsupervised exercise session per week to achieve the goal of three ST sessions per week. Thereafter, participants were asked to continue their twice weekly supervised and one unsupervised ST sessions for an additional four months. Further details of the program, including exercises and intensity prescribed, are described elsewhere [20].

### Enhanced intervention

In addition to the Standard *Lift for Life*® program, the EST intervention group (*n* = 162) also received motivationally-tailored behavioral counselling and print information. This included instructional newsletters at 2-, 4- and 6-months, covering tips on overcoming barriers to physical activity, motivation and providing variety in training; motivational incentives (at 0- and 2-months), including a sports bag, drink bottle; and behavioral telephone counselling based on principles of the stage of readiness for behavior change [11] and SCT [16]. Telephone counselling was conducted regularly (progressing from weekly, to fortnightly, to monthly to bi-monthly) for 6-months by a psychology-qualified researcher. In each counselling session, participants’ stage of change for ST was determined as well as their physical activity beliefs, barriers, motivations and goals. Counselling was then tailored to the participants’ stage of change, with on-going assessment of progression towards goals and the provision of encouragement and assistance (i.e. promoting self-efficacy and rewards/incentives) in overcoming barriers to physical activity.

### Measures

All measures were undertaken by trained research staff and were performed at baseline, 6- and 12-months, unless stated.

### ST adoption and maintenance

ST participation was assessed at 2-, 4-, 6-, and 12-months using a modified version of the CHAMPS instrument, a reliable and valid self-report, self-completion survey of physical activity for older adults [21, 22]. The CHAMPS instrument was modified by only including questions specific to ST. Further, the questions in the modified version specified the context of the ST (supervised and un-supervised) as well as the setting (gym or home) in which it was undertaken. In accordance with the recommended goals of the *Lift for Life*® program adoption was defined as undertaking three or more weekly ST sessions at each of the assessments during the first 6-months and maintenance was defined as completing three or more sessions at each of the assessments until 12-months.

### Anthropometric and glycemic assessments

Blood samples were collected at a local pathology centre for the determination of glycated haemoglobin levels (HbA1c). One pathology service with multiple collection centres was used for all HbA1c analyses. HbA1C was assessed using the Roche Tina-quant Hemoglobin A1c Gen 2 method performed on a Roche Integra 800. Anthropometric measurements, including body weight and height were assessed at 2-, 4-, 6-, and 12-months using standardized protocols [23].

### Muscle strength

Under the supervision of a trainer, muscle strength of the upper body (chest/shoulders) and lower body (quadriceps) was assessed using the three-repetition maximum strength (3-RM) [24] test for the bench press and leg press, respectively. The 3-RM is the heaviest weight that an individual can complete three repetitions for each exercise whilst maintaining correct form. Both these exercises have been used frequently in the literature to provide an indication of strength in the large muscle groups of the body (e.g. pectorals, bench press; quadriceps, leg extension).

### Demographic characteristics and leisure-time physical activity (LTPA)

Age, sex, current medication use (dose, frequency, duration and reason for the prescription), education, employment status and smoking status were assessed using a self-report survey. A modified version of the Active Australia Survey [25], a widely used self-report measure with acceptable reliability and validity [26], was used to assess changes in overall LTPA at 2-, 4-, 6-, and 12-months. The Active Australia Survey was modified by asking participants to report their LTPA excluding strength training.

### Statistical analysis

Statistical analyses were conducted using SPSS software version 19.0 and Stata statistical software release 12.0 (Stata College Station, TX). Analyses were undertaken for the pooled sample and separately for those with and without T2DM. A binary outcome was defined as adherence to the recommended guideline (performing ≥ 3 ST sessions per week), which was analyzed using a random effects logistic regression with a fixed effects of time, group, interaction effect between time and group and controlling for age, sex, presence of T2DM (pooled analysis) at baseline and BMI. The outcome measures of HbA1c, upper and lower body muscle strength, weight, and LTPA were analyzed as continuous variables using a normal linear mixed model fixed effects of time, group and a group-by-time interaction controlling for age, sex, presence of T2DM (pooled analysis) and BMI. Weight was not adjusted for BMI as weight and BMI were highly correlated (correlation = 0.81). In the pooled and T2DM analyses, HbA1c was further adjusted for type of medication and change in medication from baseline to post-intervention. Both the random effects logistic and normal linear mixed models included random effects associated with both the clusters (fitness centres) and the units of analysis (participants) to take the clustered structure of the data into account and to allow the residuals associated with the longitudinal measures on the same unit of analysis to be correlated. LTPA (minutes per week) was calculated by summing the time spent in walking and moderate activity and twice the time spent in vigorous activity (not including gardening and yard work) [25]. Data were truncated at 1680 min per week. This variable was transformed by the natural log to yield normally distributed residuals. For this variable, results were reported as percent change.

Estimated interactions were presented as the net absolute or percent difference in change between the EST and the SST group. For adherence, the size of the random effects were reported as the median odds ratio to indicate how large the variation was between two randomly chosen participants from both the same fitness centre and a different fitness centre [27]. The analyses used the intention to treat principle, with participants analysed according to the initial randomized assignments. The last observation carried forward method was implemented to impute missing data of covariates only.

## Results

A total of 38 participants (SST, *n* = 19; EST, *n* = 19) withdrew from the study during the adoption phase (0-6 months). A further five participants (SST, *n* = 1; EST, *n* = 4) withdrew during the maintenance phase (6-12 months). Reasons for withdrawal are provided in Fig. 1. Data from a total of 318 participants was included in the final analyses. With the exception of HbA1c levels and the proportion of participants taking hypoglycemic medication, which were higher in the EST group, no other between-group differences were observed at baseline (Table 1).

**Table 1:** Descriptive characteristics of the Standard and Enhanced strength training groups at baseline

	Standard	Enhanced
*N*	156	162
*Women, n (%)*	90 (58 %)	97 (60 %)
*Age (years)*	55.5 ± 8.6	56.3 ± 8.7
*Diagnosed type 2 diabetes, n (%)*	49 (31 %)	68 (42 %)
*Current Smokers, n (%)*	5 (3)	8 (5 %)
*Education*		
Did not complete high school, n (%)	26 (17 %)	24 (15 %)
Completed high school, n (%)	30 (20 %)	32 (21 %)
Technical/trade qualification, n (%)	19 (12 %)	23 (15 %)
University/tertiary, n (%)	77 (51 %)	77 (49 %)
*Employment status*		
Full time, n (%)	59 (48 %)	54 (43 %)
Part time, n (%)	42 (34 %)	33 (26 %)
Home duties, n (%)	3 (3 %)	5 (4 %)
Retired, n (%)	11 (9 %)	21 (17 %)
Other, n (%)	7 (6 %)	12 (10 %)
*Anthropometry*		
Height (cm)	167.5 ± 8.7	166.2 ± 10.0
Weight (kg)	93.4 ± 17.3	93.1 ± 19.5
BMI (kg/m^2^)	33.2 ± 5.4	33.7 ± 6.7
*HbA1c (%)* ª	6.0 ± 0.7	6.3 ± 1.0^*^
*Muscle strength*		
Upper body (kg) ^b^	27 ± 16	29 ± 14
Lower body (kg) ^c^	109 ± 52	117 ± 50
*Leisure-time physical activity (mins/week)* ^d^	205 ± 237	198 ± 259
*Medication*		
Oral hypoglycemic medication use, *n (%*)	21 (14 %)	43 (27 %)^*^
Insulin use, n (%)	2 (1 %)	2 (1 %)
Both, n (%)	7 (5 %)	4 (3 %)
*Change in medication during between 0–6 months*		
Increased, n (%)	1 (4 %)	6 (13 %)
Decreased, n (%)	3 (12 %)	2 (4 %)
Same, n (%)	21 (84 %)	38 (83 %)
*Change in medication during between 6–12 months*		
Increased, n (%)	3 (17 %)	4 (10 %)
Decreased, n (%)	0 (0 %)	3 (8 %)
Same, n (%)	15 (83 %)	33 (82 %)

Data are n (%) or means ± Standard deviation

^*^ *p* < 0.05 compared to Standard ST group

ª 8 participants were missing their baseline HBA1c levels

^b^5 participants were missing their baseline upper body muscle strength scores

^c^13 participants were missing their baseline lower body muscle strength scores

^d^16 participants were missing their baseline leisure-time physical activity scores

### ST Adoption and maintenance

#### Pooled analysis

**Fig 2:**
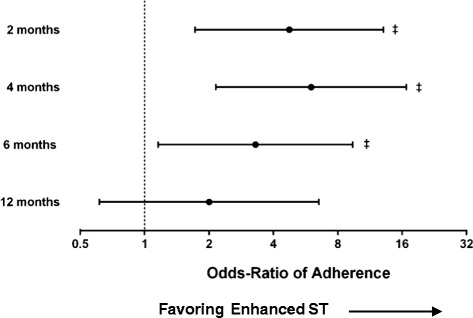
Forrest plot showing odds ratio of adherence to strength training at 2-, 4-, 6- and 12- months*. * based on the mixed model with random effects; Adherence = ≥3 sessions per week. ‡ *p* < 0.05 difference between the Enhanced and Standard ST group

In the adoption phase, 127 (40 %) participants completed 3 sessions of ST per week (SST, *n* = 49; EST, *n* = 78). The odds ratio (OR) of adopting ST were 3.3 times (95 % CI 1.2 to 9.4) higher for participants in the EST compared to SST group. At 12-months, the number of participants maintaining ST (3 sessions/week) was reduced to 47 (15 %; SST, *n* = 15; EST, *n* = 32), with the OR of maintaining the program greater (but not significant) for the EST compared to SST group (OR 2.0, 95 % CI 0.6 to 6.5) (Fig. 2). The median odds ratio (MOR) of adherence between two randomly selected participants from the same leisure centre was 1.7 (95 % CI 1.4 to 2.1). The MOR between two randomly selected participants from two different centres was 1.8 (95 % CI 1.5 to 2.3). The between-persons variation in adherence odds was of the same order of magnitude as the intervention effect, indicating that the intervention effect was quite substantial.

#### Stratified analyses

In those with T2DM, the odds of adopting ST were higher for the EST group relative to the SST group (OR 8.2, 95 % CI 1.5 to 45.5). In those without T2DM, the odds of adopting ST were higher for the EST group but this was not significant (OR 2.7, 95 % CI 0.9 to 7.9). No significant differences between groups were observed during the maintenance phase in those with T2DM and those without T2DM.

### Changes in glycemic control

#### Pooled analysis

Relative to baseline, HbA1c did not change in the SST group but decreased in the EST group during the adoption phase (0–6 months), which resulted in a significant group-by-time interaction and net difference for the change of -0.13 % (*P* < 0.05) (Table 2). At 12-months there was a greater reduction in HbA1c in the EST group compared with the SST group (-0.17 %, *P* < 0.05). Similar findings were seen in models adjusted for type and change in medication.

**Table 2:** Estimated mean net difference between the Standard and Enhanced strength training groups following the adoption and maintenance phases and after 12 months

	Adjusted estimated mean net difference for the change between the enhanced and standard ST group (95 % CI) – pooled data			Adjusted estimated mean net difference for the change between the enhanced and standard ST group (95 % CI) for participants with T2DM		
	Adoption	Maintenance		Adoption	Maintenance	
Outcome variables	0–6 month	6–12 months	0–12 months	0–6 month	6–12 month	0–12 month change
HbA1c (%)	−0.13 (-0.26, -0.01) ‡	−0.04 (-0.18, 0.10)	−0.17 (-0.30, -0.05) ‡	−0.3 (-0.6, -0.004) ‡	−0.1 (-0.5, 0.2)	−0.4 (-0.7, -0.1) ‡
BMI (kg/m^2^)	−0.1 (-0.3, 0.1)	−0.3 (-0.5, -0.1) ‡	−0.4 (-0.6, -0.2) ‡	−0.3 (-0.6, 0.1)	−0.3 (-0.7, 0.1)	−0.6 (-0.9, -0.2) ‡
Weight (kg)	−0.3 (-0.9, 0.3)	−0.7 (-1.4, -0.1) ‡	−1.0 (-1.6, -0.4) ‡	−0.9 (-1.9, 0.1)	−0.7 (-1.8, 0.3)	−1.6 (-2.6, -0.6) ‡
Upper body strength (kg)	−0.9 (-3.0, 1.3)	−1.6 (-4.0, 0.7)	−2.6 (-4.7, -0.3) ‡	−1.7 (-5.3, 2.0)	−1.9 (-6.1, 2.3)	−3.6 (-7.5, 0.4)
Lower body strength (kg)	−0.5 (-10.2, 9.1)	−0.9 (-11.4, 9.6)	−1.4 (-11.6, 8.8)	−3.9 (-20.9, 13.1)	0.2 (-18.4, 18.8)	−3.7 (-22.0, 14.6)
Leisure-time physical activity (%)	38 (-10, 110)	−18 (-48, 29)	13 (-27, 74)	39.9 (-36.1, 206.5)	−26.1 (-68.7, 74.8)	3.3 (-55.0, 137.4)

Data are mean net difference (95 % CI). Mean estimated net difference for the change between groups represents the within-group change in the Enhanced group minus the within-group change in the Standard group, adjusted for confounders

‡*p* < 0.05 group-by-time interaction for the between-group difference for the change relative to baseline or 6 -months

#### Stratified analyses

For participants with T2DM, the EST group showed a significant reduction in HbA1c at 6-months and 12-months. The adjusted between group differences was -0.3 % (*P* < 0.05) during the adoption phase and -0.4 % (*p* < 0.05) at 12-months. There were no between group differences in HbA1c during both adoption and maintenance phase in participants without T2DM.

### Changes in weight and BMI

#### Pooled analysis

For weight and BMI, there were similar significant reductions in both groups relative to baseline after the adoption phase, but at 12-months the EST group experienced a greater reduction in weight compared to the SST group (group-by-time interaction, *P* < 0.05).

#### Stratified analyses

Among participants with T2DM, the adjusted between-group differences at 6-months approached significance (-0.9 kg, *P* = 0.08) for weight. Similarly at 12-months there was a greater reduction in weight (-1.6 kg, *p* < 0.01) and BMI (-0.6 kg/m^2^, *p* < 0.05) in the EST group. No between group differences in weight were observed during adoption or maintenance phase for participants without T2DM.

### Changes in muscle strength and LTPA

#### Pooled analysis

There was an increase in upper body strength (bench press) (9.7 kg, 95 % CI: 8.2 to 11.2) in the SST group and in the EST group (8.8 kg, 95 % CI: 7.3 to 10.3) during the adoption phase. Significant increases were also seen in the lower body muscle strength (leg press) during the adoption phase (37.8 kg, 95 % CI: 30.9 to 44.7) in the SST group and (37.3 kg, 95 % CI: 30.5 to 44.0) in the EST group. These benefits were maintained at 12-months from baseline. However, from baseline to 12-months the change in upper body muscle strength was lower in the EST group compared to the SST group (net difference, -2.6 kg, 95 % CI: -4.7 to -0.3). The results for all outcomes remained unchanged after adjusting for time spent in LTPA. There were no significant between-group differences in LTPA.

#### Stratified analyses

There was a significant increase in upper and lower body strength and LTPA within the SST and EST groups at 6- and 12-months from baseline for participants both with and without T2DM. However, no significant between group changes from baseline were observed in upper body strength, lower body strength and LTPA during the adoption or the maintenance phase for participants with T2DM and participants without T2DM.

## Discussion

A community-based EST program undertaken by overweight adults and those with T2DM that included additional support such as instructional newsletters, motivational incentives and behavioral telephone counselling was more effective for the adoption, but not the maintenance, of ST. Furthermore, in those with T2DM, the EST program led to small, but significantly greater improvements in glycemic control and a modest change in body weight relative to the SST program at 12-months. In contrast, there were no differences for the change in lower body muscle strength between the two groups during either phase.

The finding that the EST program incorporating motivationally-tailored behavioral counselling and print information significantly increased adoption to the ST program is consistent with previous studies that have shown favourable results in terms of physical activity participation in the early or initiation phases [28]. Notwithstanding the potential limitation of having insufficient power in the current study to detect differences in maintenance, it could also be speculated that since the maintenance period included minimal contact (i.e. phone calls and incentives were discontinued after the initial 6-month adoption phase) [28], it is possible that ST had not become habitual. Indeed, providing additional follow-up contact and prompts beyond 6-months may be necessary for achieving long-term maintenance in physical activity interventions [17, 29].

Another potential explanation for the findings for maintenance may relate in part to the theoretical framework used to develop intervention strategies. SCT posits that personal factors (e.g. self-efficacy and outcome expectations) and environmental factors (e.g. social support, environmental supports) predict physical activity behavior change [16]. However, this theory has been criticized for its lack of distinction between factors such as self-efficacy and social support that promote adoption, and those that promote maintenance of behavior change [17]. Previous research has suggested that self-efficacy and outcome expectations are more important for behavioral adoption, whilst social support is more important for behavioral maintenance [17]. However, the current study utilized behavioral telephone counselling (social support) during the adoption phase but not during the maintenance phase. Thus, it may be that such support strategies needed to be continued or switched for other forms of behavioral support such as text messaging or emails in order to enhance the maintenance of ST.

Recent meta-analyses of randomized controlled trials investigating the effects of resistance training on HbA1c levels in adults with T2DM or obesity-related impaired glucose metabolism reported mean absolute reductions in HbA1c in the magnitude 0.48 to 0.60 % relative to controls [30, 31]. The HbA1c change observed in our study in those with T2DM was comparable to what was observed in four of the 10 studies included in the meta-analysis and is similar to the differences we have previously reported following centre-based ST for 14 months in people with T2DM [2]. From a clinical perspective, this represents a small absolute change; however, it is important to acknowledge that in contrast to the SST program, the EST intervention was effective for preventing a deterioration in glycemic control during the 12-month study period, which is consistent with our previous investigation after 6-months of gymnasium-based followed by 6-months home-based training in individuals with T2DM [32]. Furthermore, a recent meta-analysis [33] found that structured exercise training of more than 150 min per week was associated with greater HbA1c declines than that of 150 min or less per week. Thus, it is possible that the reduced adherence to the recommendations of three or more sessions per week during the maintenance phase may have lessened the impact of the intervention on glycemic control due to the consequent reduction in overall training volume (amount of weight lifted/number of repetitions and sets performed per week) during this phase.

Key strengths include the cluster randomized design, the duration of the observation period and the focus on a community-based intervention setting. Since very little research has been conducted on the long-term effectiveness, retention and adherence of ST programs in overweight adults or those with T2DM [34] these findings provide important new insights to inform the practicalities, sustainability and viability of methods to safely implement ST programs at the population level. Alterations in medication dosage in those with T2DM during the intervention may have altered the ability to detect changes in glycemic control produced by strength training, however, changes in medication, as reflected by self-report of the addition or removal of medication, were similar between groups, and the results were still evident after adjustment for medication changes. Other limitations relate to the use of self-report measures of resistance training and LTPA, which may be subject to bias and recall difficulties and the absence of specific measures of fat and muscle mass. Finally, adverse events were not documented throughout the study.

In summary, an EST program incorporating motivationally-tailored behavioral strategies was more effective than a SST program for the adoption of ST over an initial 6-month period in a community-based health and fitness facility, in overweight adults and those with T2DM. Additionally, the initial 6-month EST program led to significant, albeit modest improvements in glycemic control compared to the SST program, which was maintained following the maintenance phase. This finding, combined with the reduced adherence to ST recommendations during the maintenance phase, suggests that further research is needed to determine the factors that can optimize long-term exercise adherence in the community setting in this population and enhance the training-induced changes in glycemic control.
